# The synergistic impact of Universal Health Coverage and Global Health Security on health service delivery during the Coronavirus Disease-19 pandemic: A difference-in-difference study of childhood immunization coverage from 192 countries

**DOI:** 10.1371/journal.pgph.0003205

**Published:** 2024-05-10

**Authors:** Sooyoung Kim, Tyler Y. Headley, Yesim Tozan

**Affiliations:** 1 Department of Public Health Policy and Management, School of Global Public Health, New York University, New York, New York, United States of America; 2 New York University Abu Dhabi, Abu Dhabi, United Arab Emirates; 3 Department of Global and Environmental Health, School of Global Public Health, New York University, New York, New York, United States of America; PLOS: Public Library of Science, UNITED STATES

## Abstract

Universal Health Coverage (UHC) and Global Health Security (GHS) are two high-priority global health agendas that seek to foster health system resilience against health emergencies. Many countries have had to prioritize one agenda over the other due to scarce resources and political pressures. To aid policymakers’ decision-making, this study investigated the individual and synergistic effects of countries’ UHC and GHS capacities in safeguarding essential health service delivery during the COVID-19 pandemic. We used a quasi-experimental difference-in-difference methodology to quantify the relationship between 192 countries’ progress towards UHC and GHS and those countries’ abilities to provide 12 essential childhood immunization services between 2015 and 2021. We used the 2019 UHC Service Coverage Index (SCI) to divide countries into a “high UHC group” (UHC SCI≥75) and the rest (UHC SCI 75), and similarly used the 2019 GHS Index (GHSI) to divide countries into a “high GHS group” (GHSI≥65) and the rest (GHSI<65). All analyses were adjusted for potential confounders. Countries with high UHC scores prevented a 1.14% (95% CI: 0.39%, 1.90%) reduction in immunization coverage across 2020 and 2021 whereas countries with high GHSI scores prevented a 1.10% (95% CI: 0.57%, 1.63%) reduction in immunization coverage over the same time period. The stratified DiD models showed that across both years, high UHC capacity needed to be augmented with high GHS capacity to prevent a decline in immunization coverage while high GHS alone was able to safeguard immunization coverage. This study found that greater progress towards both UHC and GHS capacities safeguarded essential health service delivery during the pandemic but only progress towards GHS capacity was both a necessary and likely sufficient element for yielding this protective effect. Our results call for strategic investments into both health agendas and future research into possible synergistic effects of the two health agendas.

## Introduction

The ongoing Coronavirus Disease 2019 (COVID-19) pandemic has underscored the urgent need to strengthen the global health architecture for pandemic prevention, preparedness, and response [1]. Within the context of increasing health systems resilience, Universal Health Coverage (UHC) and Global Health Security (GHS) are two key global health agendas that are highly prioritized by global and national policy bodies, including the World Health Organization, and seek to foster strong health systems for a healthier and safer world [2]. There is a clear link between the two agendas: progress towards UHC promotes GHS by ensuring widespread access to comprehensive and inclusive health services before, during, and after public health emergencies [3–5]. Despite the interplay between UHC and GHS, there has been a long-standing disconnect between the two agendas at the policy level, and country-level efforts to align and integrate policies and funding activities have been limited due to scarce resources and domestic and global political pressures [2, 5]. Due to these impediments, few countries have strategically prioritized policies that are aligned with both frameworks or have invested in integrated health systems, which simultaneously support UHC and GHS capacities [5]. The dearth of concurrent investment into both agendas may have led to worse health outcomes during the pandemic; some scholars have argued that countries which prioritized UHC and GHS integration better mitigated the pandemic’s impacts and might recover from COVID-19 more quickly [5]. The COVID-19 pandemic’s varied impacts on health systems have highlighted the importance of UHC as a crucial foundation of pandemic preparedness and response [1]. Countries’ progress towards UHC requires not only overall health systems strengthening but also sustainable pre-pooled funding mechanisms [6]; countries with strong UHC systems should therefore be more resilient to external shocks and more agile when responding to public health emergencies [2]. Consistent with this argument, studies investigating public health capacity showed that the lack of adequate primary health care compromised countries’ ability to implement an equitable response to COVID-19 or safeguard health services delivery [7, 8]. In a recent study, using childhood immunization coverage data from 192 countries, we similarly showed that countries with greater progress towards UHC were associated with a significantly smaller decline in vaccine coverage during the first year of the COVID-19 pandemic [9].

An ongoing debate has been about whether investments into or the prioritization of one health framework alone, whether UHC or GHS, may result in optimal health system preparedness or response capacity, or whether a combination of investments towards both agendas is necessary to yield maximal health system resilience against pandemics and other health emergencies. To this end, some scholars have argued that investments in core GHS capacities (i.e., surveillance, risk communication, and coordination) alone are insufficient for comprehensive pandemic preparedness and response; these scholars instead advocate for concurrent investments in GHS and UHC [1]. This argument appears to be borne out by initial studies on the relationship between GHS and health system resilience, which found that GHS had null [10–12], mixed [13], or even negative [14] associations with countries’ ability to counter and withstand the COVID-19 pandemic. Given the suboptimal nature of investing into just one framework rather than both simultaneously, several international calls have been made for integrating GHS into UHC objectives to protect both individual and population health and to prevent health system failures during health emergencies [5]. In response to these calls, multiple international initiatives including proposals for a pandemic treaty and a pandemic fund have been launched to better prepare for and respond to the current pandemic and future health emergencies [1, 15]. Despite growing calls for the alignment of the two health frameworks, there is no empirical evidence on the potential synergistic effects of UHC and GHS on safeguarding population health before, during, or after a public health crisis. While the ongoing COVID-19 pandemic provides an unprecedented opportunity to improve our understanding of the effects of UHC and GHS on health systems strengthening, extant studies examining their impact are limited in number, assessed the potential role of UHC and GHS individually, or generally focused on COVID-19 outcomes during the first wave of the pandemic in early 2020 [10–14, 16–18].

Building and expanding on our previous study [9], this study examined the individual and synergistic protective effects of UHC and GHS on childhood vaccination coverage during the first two years of the COVID-19 pandemic (2020–2021). Our main hypothesis was that countries’ greater progress towards UHC and/or GHS capacities enable countries’ health systems to be more resilient to external shocks, such as the COVID-19 pandemic. This resilience will positively affect countries’ abilities to provision basic and essential public health services, including routine childhood immunizaitons. Childhood vaccination coverage is used as the outcome measure because immunization is considered an essential health service across all health care settings and all countries report annual vaccine coverage data [19]. To assess the protective effects of UHC and GHS on vaccination coverage individually, we employed a difference-in-difference (DiD) design following the methods we used in our previous study. We operationalized the UHC Service Coverage Index (SCI) 2019 as a measure of countries’ progress towards UHC and the Global Health Security Index (GHSI) as a measure of countries’ capacity for pandemic preparedness and response. To quantify the synergistic protective effects of UHC and GHS capacities on vaccination coverage during the same time period, we employed a stratified DiD approach. We hypothesized that countries with greater progress toward UHC, as represented by higher UHC SCI 2019 values, and stronger GHS capacity, as represented by higher GHSI scores, would better safeguard countries’ ability to provide essential health services during the COVID-19 pandemic. The findings of this study have the potential to inform local, national, and global policymakers when setting priorities and making investment decisions to strengthen countries’ health system readiness and resilience against future public health emergencies.

## Methods

We employed a quasi-experimental difference-in-difference (DiD) design to quantify the independent and synergistic protective effects of UHC and GHS capacities on countries’ abilities to safeguard their provision of essential childhood immunization services during the COVID-19 pandemic. The DiD design has also been used to measure the pandemic’s effects on neonatal outcomes [20, 21] and healthcare utilization rates [22]. The benefits of using childhood immunization coverage as a proxy to measure the effect of COVID-19 on health system resilience are two-fold: first, immunization is considered an essential health service across all healthcare settings [19]. While most studies have looked at the COVID-19 cases and mortality rates to elucidate overall health system resilience, this approach cannot parse apart the significant confounding effects of countries’ surveillance and laboratory test capacities, socio-behavioral factors influencing population testing behaviors, and population age structures and underlying morbidity factors [23]. Analytical frameworks measuring the impact of the pandemic on overall health system resilience through other routine but essential health services like childhood immunization programs can, therefore, better illustrate the direct impact of COVID-19 on countries’ health systems without severe concerns about the role of confounding variables. Second, the data availability and generalizability of other indicators of essential health services, especially over time, have either varied during the pandemic or did not exist prior to the pandemic; this makes the application of causal inference methods difficult. Childhood immunization rates, however, are a robust indicator both over time and at the global level because all countries have national immunization programs, and this data has been annually aggregated, reported, and standardized for decades [19].

### Data

Our dependent variable on national childhood immunization rates was derived from the WHO/UNICEF Joint Estimates of National Immunization Coverage [24]. The data includes the annual vaccination coverage rates of 14 vaccines for 195 countries spanning the years 1997 to 2021. We excluded the yellow fever vaccine (YFV) from our analysis because it is not administered widely across all countries, and thus leads to a data imbalance. We also excluded data on the first dose of the inactivated polio vaccine (IPV-1) because IPV-1 data was not available across all years under observation. After these exclusions, our analysis included 12 different childhood vaccines: Bacille Calmette-Guérin (BCG); the first and third dose of diphtheria, tetanus toxoid, and pertussis containing vaccine (DTP1, DTP3); the birth dose of hepatitis B vaccine (HEPB-3); the third dose of hepatitis B containing vaccine (HEPBB); the third dose of *Haemophilus influenzae* type B containing vaccine (HIB3); the first and second doses of measles containing vaccine (MCV1, MCV2); the third dose of pneumococcal conjugate vaccine (PCV3); the third dose of polio containing vaccine (POL3); the second or third dose of rotavirus vaccine (ROTAC); and the first dose of rubella containing vaccine (RCV1). Our primary independent variables were GHSI 2019 [25], which was developed by a partnership between the Nuclear Threat Initiative (NTI), the Johns Hopkins Center for Health Security at the Bloomberg School of Public Health, and the Economist Impact, and the UHC SCI 2019, which was obtained from the Institute for Health Metrics and Evaluation (IHME) [26]. GHSI 2019 is an assessment of countries’ health security and related capabilities necessary to prepare for future outbreaks including epidemics and pandemics [25]. GHSI 2019 consists of 37 indicators across 6 categories—prevention, detection and reporting, rapid response, health system, compliance with international norms, and risk environment. UHC SCI 2019 is a robust and widely used index that measures countries’ effective service coverage [26]. The UHC index is a weighted aggregate of 23 indicators measuring service coverage across the full spectrum of essential health service coverage—promotion, prevention, treatment, rehabilitation, and palliation—and across five age groups—newborn, children under 5 years, children and adolescents between 5–19 years, adults between 20–64 years, and older adults age 65 years or more—across the life course. Both indexes range from 0 to 100, with 100 indicating the highest preparedness capacity or a higher effective health service coverage. We also used the World Bank’s income level classification to assign countries to high, upper-middle, lower-middle, or low income groups [27]. Countries which lacked data on income classification or UHC/GHS scores were dropped from this analysis (Cook Island, Niue, Palestine), resulting in 192 included countries.

### Statistical analysis

We first tested for the individual effects of countries’ progress toward UHC and GHS capacities on vaccination coverage using a quasi-experimental DiD design. We divided countries into treatment and control groups based on their progress toward UHC and GHS capacities using their respective index scores (UHC SCI and GHSI 2019). While both indexes are designed to summarize countries’ progress towards certain policy agendas on a standardized scale of 0 to 100, there is a dearth of prior studies identifying a threshold index value to define sufficient progress. Therefore, we first tested different UHC SCI and GHSI 2019 cutoff values to determine a meaningful delineator between the treatment and control groups. For GHSI 2019 (Fig A in S1 Text for its distribution), we performed DiD analyses using a sliding scale of cutoff values between 40 (53rd percentile) and 80 (99th percentile) with step increments of 5 to evaluate the lowest threshold value of GHSI 2019 where significant resilience occurred, as demonstrated by a statistically significant DiD estimator. Similarly, for UHC SCI 2019 (Fig B in S1 Text for its distribution), we used a sliding scale of values between 60 (53^rd^ percentile) and 90 (92^nd^ percentile) with step increments of 5 to observe the lowest threshold value when significance occurred. We then used these threshold values to define the treatment and control groups in subsequent analyses given the policy importance of understanding when and to what extent a significant positive effect is observed.

To perform DiD analyses, we used the WHO/UNICEF Joint Estimates of National Immunization Coverage data of 12 vaccines per given year and country as our dependent variable. We leveraged the COVID-19 pandemic to define a pre-post period, wherein the years prior to 2020 were defined as pre-pandemic, and the pandemic years of 2020–2021 were defined as post years (i.e., during the pandemic). We used doubly-robust DiD estimation methods proposed by Sant’Anna and Zhao [28] to obtain the average treatment effect on the treated (ATT) of higher levels of UHC or GHS capacities in safeguarding immunization coverage during the COVID-19 pandemic. The binary *Treatment* variable represents countries’ assigned group based on their progress towards UHC or GHS. We included time fixed effects using calendar years, as represented by *γ*_*t*_, and controlled for the group fixed effects using country and vaccine type, denoted by *α*_*i*_.


$$(Immunization\;Coverage_{i,t})=\beta_{0}+\beta_{1}*Treatment_{i,t}+\gamma_{t}+\alpha_{i}+u_{i,t}$$ (Eq 1)

In all analyses, we included as covariates countries’ income group as per the World Bank classification and countries’ geographical region as per the WHO regional classification. For all analyses, we checked whether the parallel pre-trend assumption was satisfied after controlling for pre-trend covariates for each model. As a result of this process, our final analytical sample only included the data collected from 2015 onwards.

We next examined whether there was a synergistic impact of a country’s progress towards UHC or GHS capacities on immunization service provisioning during the pandemic by cross-tabulating the two treatment variables to further divide the countries into four separate groups: “High UHC/High GHS,” “High UHC/Low GHS,” “Low UHC/High GHS,” and “Low UHC/Low GHS.” We then performed additional DiD analyses to compare the first three groups to countries with low progress towards UHC and GHS. Acknowledging that DTP3 and MCV1 are part of the 23 indicators constituting UHC SCI 2019, we computed the correlation between UHC SCI 2019 and the overall vaccination coverage rate, DTP3 rate, and MCV1 rate, respectively, and observed no significant correlation or colinearity. We also repeated our analyses by excluding DTP3 and MCV1 from our independent variables and observed no significant impact in the coefficients of interest and their statistical significance (Figs E and F and Tables I~M in S1 Text).

All analyses were conducted using R software (Version 4.0.3; S1 Text). A Strengthening the Reporting of Observational Studies in Epidemiology (STROBE) checklist [29] is included in the Supplementary Materials (Table A in S1 Text). Replication data and code are available at https://github.com/sk9076/UHC_GHS_2021.

## Results

Our dataset included 16,205 observations spanning 192 countries from 2015 to 2021 that satisfied the parallel pre-trend assumption. A total of 4,630 observations (28.6%) took place during each of the pandemic years of 2020 and 2021, whereas 11,575 (71.4%) took place from 2015 to 2019. When we tested the different cutoff values to divide the countries into treatment and control groups, the lowest cutoff value with a significant effect in safeguarding a decline in immunization coverage rates during the pandemic was 75 for UHC and 60 for GHSI (Figs C and D and Tables B and C in S1 Text). A complete list of the countries included in the analysis stratified by UHC and GHSI scores is presented in Table 1.

**Table 1:** Countries grouped by UHC and GHS scores.

Countries with UHC SCI 2019≥75 and GHSI≥60 (N = 17)	Denmark, Finland, France, Portugal, Republic Of Korea, Slovenia, Sweden, Australia, Belgium, Canada, Germany, Netherlands, Norway, Spain, Switzerland, United Kingdom, United States
Countries with UHC≥75 and GHSI<60 (N = 23)	Austria, Costa Rica, Croatia, Czechia, Estonia, Greece, Ireland, Israel, Italy, Japan, Kuwait, Malta, Monaco, Peru, Qatar, Singapore, Slovakia, Andorra, Cyprus, Iceland, Luxembourg, New Zealand, San Marino
Countries with UHC<75 and GHSI≥60 (N = 3)	Latvia, Malaysia, Thailand
Countries with UHC<75 and GHSI<60 (N = 149)	Afghanistan, Albania, Algeria, Angola, Argentina, Armenia, Azerbaijan, Bangladesh, Belarus, Belize, Benin, Bhutan, Bolivia (Plurinational State Of), Bosnia And Herzegovina, Botswana, Brazil, Brunei Darussalam, Bulgaria, Burkina Faso, Burundi, Cabo Verde, Cambodia, Cameroon, Central African Republic, Chad, Chile, China, Colombia, Comoros, Congo, Cote D’ivoire, Cuba, Democratic People’s Republic Of Korea, Democratic Republic Of The Congo, Djibouti, Dominica, Dominican Republic, Ecuador, Egypt, El Salvador, Equatorial Guinea, Eritrea, Eswatini, Ethiopia, Fiji, Gabon, Gambia, Georgia, Ghana, Guatemala, Guinea, Guinea-Bissau, Guyana, Haiti, Honduras, Hungary, India, Indonesia, Iran (Islamic Republic Of), Iraq, Jamaica, Jordan, Kazakhstan, Kenya, Kiribati, Kyrgyzstan, Lao People’s Democratic Republic, Lesotho, Liberia, Libya, Lithuania, Madagascar, Malawi, Maldives, Mali, Marshall Islands, Mauritania, Mauritius, Mexico, Micronesia (Federated States Of), Mongolia, Montenegro, Morocco, Mozambique, Myanmar, Namibia, Nauru, Nepal, Nicaragua, Niger, Nigeria, Oman, Pakistan, Panama, Papua New Guinea, Paraguay, Philippines, Poland, Republic Of Moldova, Republic Of North Macedonia, Romania, Russian Federation, Rwanda, Saint Kitts And Nevis, Saint Lucia, Saint Vincent And The Grenadines, Samoa, Sao Tome And Principe, Saudi Arabia, Senegal, Serbia, Seychelles, Sierra Leone, Solomon Islands, Somalia, South Africa, South Sudan, Sri Lanka, Sudan, Syrian Arab Republic, Tajikistan, Timor-Leste, Togo, Tonga, Tunisia, Türkiye, Turkmenistan, Tuvalu, Uganda, Ukraine, United Arab Emirates, United Republic Of Tanzania, Uruguay, Uzbekistan, Vanuatu, Venezuela (Bolivarian Republic Of), Viet Nam, Yemen, Zambia, Zimbabwe, Antigua And Barbuda, Bahamas, Bahrain, Barbados, Grenada, Lebanon, Palau, Suriname, Trinidad And Tobago

Table 2 summarizes the descriptive statistics of the model variables stratified by the two treatment variables. Prior to the pandemic (2015–2019), the overall average immunization coverage rate was 87.1% (SD = 15.3); for countries with high UHC and GHSI scores, the mean coverage rate was 92.5% (SD = 8.86). This was similar to the countries with high UHC and low GHSI scores (92.6%; SD 9.36; t = -0.11249, p-value = 0.910) and countries with low UHC and high GHSI scores (95.7%; SD 6.12; t = -5.278, p-value<0.001), but higher than countries with low UHC and low GHSI scores (85.5%; SD 16.3; t = 1442.4, p-value<0.001).

**Table 2:** Childhood immunization and summary statistics by UHC SCI 2019 and GHSI 2019.

	Countries with UHC SCI 2019≥ 75 and GHSI 2019≥60 (N = 17)	Countries with UHC SCI 2019≥75 and GHSI 2019<60 (N = 23)	Countries with UHC SCI 2019<75 and GHSI 2019≥60 (N = 3)	Countries with UHC SCI 2019<75 and GHSI 2019<60 (N = 149)	Total (N = 192)
UHC SCI 2019 Mean (SD)	88.8 (3.30)	85.3 (6.22)	69.3 (2.55)	53.5 (11.2)	60.7 (17.0)
GHSI Mean (SD)	70.0 (6.11)	48.0 (9.03)	66.1 (6.16)	35.3 (10.3)	40.4 (14.5)
World Bank Income Group (N, %)					
High	17 (100%)	21 (91.3%)	1 (33.3%)	21 (14.1%)	60 (31.3%)
Upper-Middle	0 (0%)	2 (8.7%)	2 (66.7%)	50 (33.6%)	54 (28.1%)
Lower-Middle	0 (0%)	0 (0%)	0 (0%)	48 (32.2%)	48 (25.0%)
Low	0 (0%)	0 (0%)	0 (0%)	30 (20.1%)	30 (15.6%)
WHO Region (N, %)	2 (11.8%)	2 (8.7%)	0 (0%)	31 (20.8%)	35 (18.2%)
Americas	13 (76.5%)	16 (69.6%)	1 (33.3%)	23 (15.4%)	53 (27.6%)
Europe	2 (11.8%)	3 (13.0%)	1 (33.3%)	19 (12.8%)	25 (13.0%)
Western Pacific	0 (0%)	2 (8.7%)	0 (0%)	19 (12.8%)	21 (10.9%)
Eastern Mediterranean	0 (0%)	0 (0%)	1 (33.3%)	10 (6.7%)	11 (5.7%)
Southeast Asia	0 (0%)	0 (0%)	0 (0%)	47 (31.5%)	47 (24.5%)
Africa	2 (11.8%)	2 (8.7%)	0 (0%)	31 (20.8%)	35 (18.2%)
Mean Immunization Coverage Rates (Mean, SD)					
2015–2019	92.5 (8.86)	92.6 (9.36)	95.7 (6.12)	85.5 (16.3)	87.1 (15.3)
2020	92.9 (7.80)	91.9 (8.68)	94.8 (10.1)	82.7 (15.7)	84.8 (15.0)
2021	92.2 (8.52)	91.6 (10.1)	93.3 (6.68)	88.8 (17.6)	83.2 (16.8)
2020–2021	92.6 (8.16)	91.8 (9.39)	94.0 (8.50)	81.7 (16.7)	84.0 (16.0)
2015–2021	92.5 (8.66)	92.3 (9.37)	95.2 (6.94)	84.4 (16.5)	86.2 (15.6)

*UHC SCI = Universal Health Coverage Service Coverage Index; GHSI = Global Health Security Index; WHO = World Health Organization; SD = Standard Deviation

**Fig 1:**
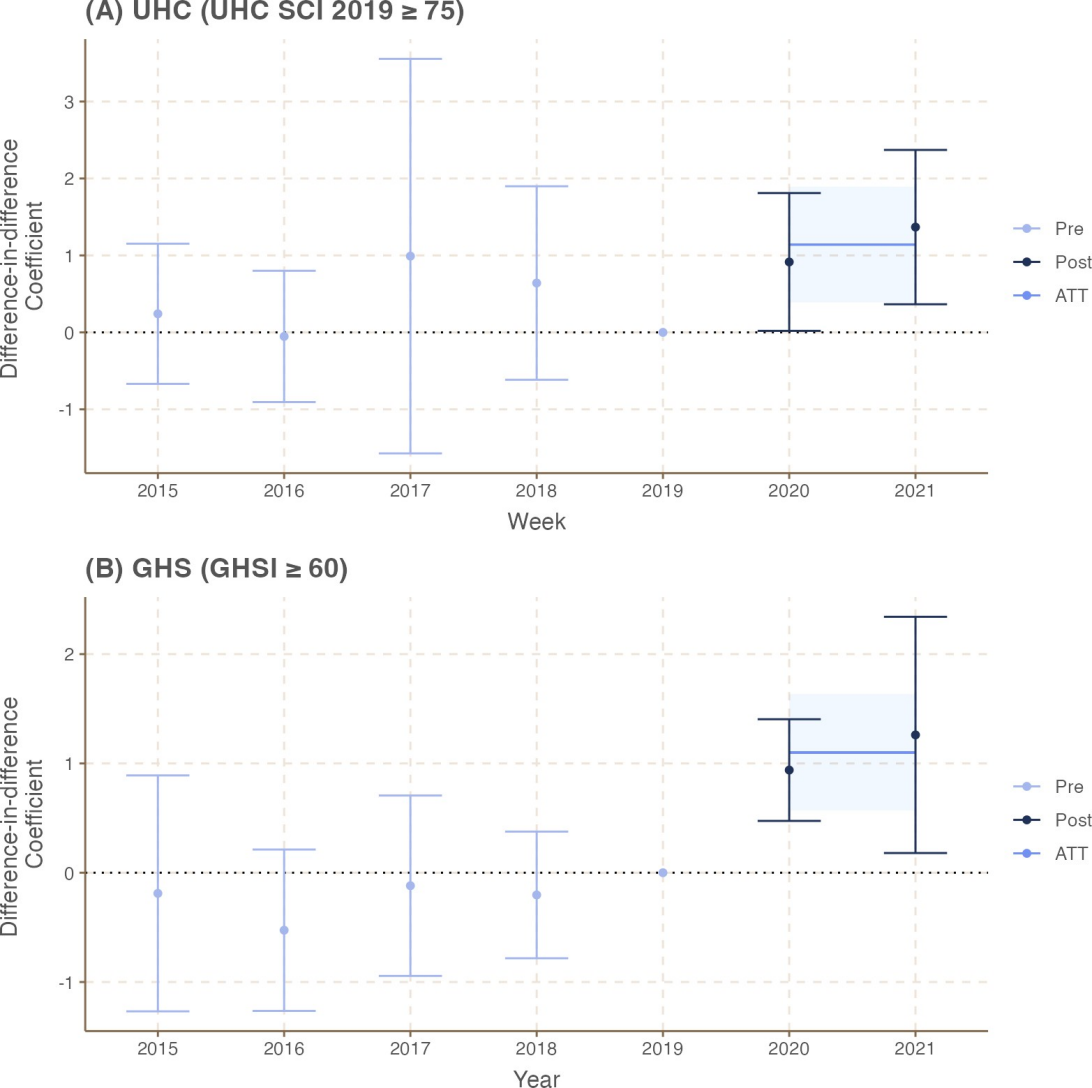
Difference-in-difference model results across 2020 and 2021.

The results of the DiD models are presented in Table 3 (Models 1 and 2) and Fig 1. The full model results (Tables C and D in S1 Text) are included in the Supplementary Materials. We found that countries with high GHS capacity (GHSI≥60) experienced a 1.100% (95% CI: 0.570%, 1.630%) reduced decline in vaccination coverage across 2021 and 2022 when controlled for the covariates. When disaggregated by year, the reduction in coverage decline was 0.939% during 2020 (95% CI: 0.474%, 1.404%) and 1.261% during 2021 (95% CI: 0.180%, 2.342%). Similarly, we found that countries’ progress towards UHC also safeguarded immunization service provision during the pandemic. Across 2020 and 2021, countries with high UHC (UHC SCI 2019≥75) prevented a 1.14% (95% CI: 0.39%, 1.90%) decline in vaccination coverage when controlled for the covariates; this effect was statistically significant in both years. The reduction in coverage decline was more pronounced in 2021 (DiD coefficient = 1.37%; 95% CI: 0.37%, 2.37%) compared to 2020 (DiD coefficient = 0.92%; 95% CI: 0.02%, 1.81%).

**Table 3:** Effect of countries’ progress towards UHC and/or GHS capacities on childhood immunization rates during the COVID-19 pandemic.

	Coefficient (95% Confidence Interval [CI])				
Variable	Model 1 (DiD for GHSI 2019≥60)	Model 2 (DiD for UHC SCI 2019≥75)	Model 3** (UHC SCI 2019≥75 and GHSI 2019≥60)	Model 4** (UHC SCI 2019≥75 and GHSI 2019<60)	Model 5** (UHC SCI 2019<75 and GHSI 2019≥60)
Overall DiD effect size (across 2020 and 2021)	1.100 (0.570, 1.630)	1.141 (0.388, 1.895)	1.946 (0.973, 2.919)	0.839 (-0.083, 1.762)	2.011 (0.421, 3.602)
DiD effect size for 2020	0.939 (0.474, 1.404)	0.915 (0.020, 1.810)	1.790 (0.699, 2.881)	0.893 (-0.007, 1.793)	2.316 (0.842, 3.790)
DiD effect size for 2021	1.261 (0.180, 2.342)	1.368 (0.365, 2.371)	2.102 (0.938, 3.266)	0.786 (-0.599, 2.171)	1.717 (-1.346, 4.759)
p-value for parallel pre-trend assumption check	0.107	0.532	0.106	0.625	0.400

* All analyses are controlled for countries’ income group, vaccine type, calendar year, and geographical region.

** Control group for DiD includes countries with UHC SCI 2019<75 and GHSI 2019<60.

UHC SCI = Universal Health Coverage Service Coverage Index; GHSI = Global Health Security Index; DiD = Difference-in-Difference.

**Fig 2:**
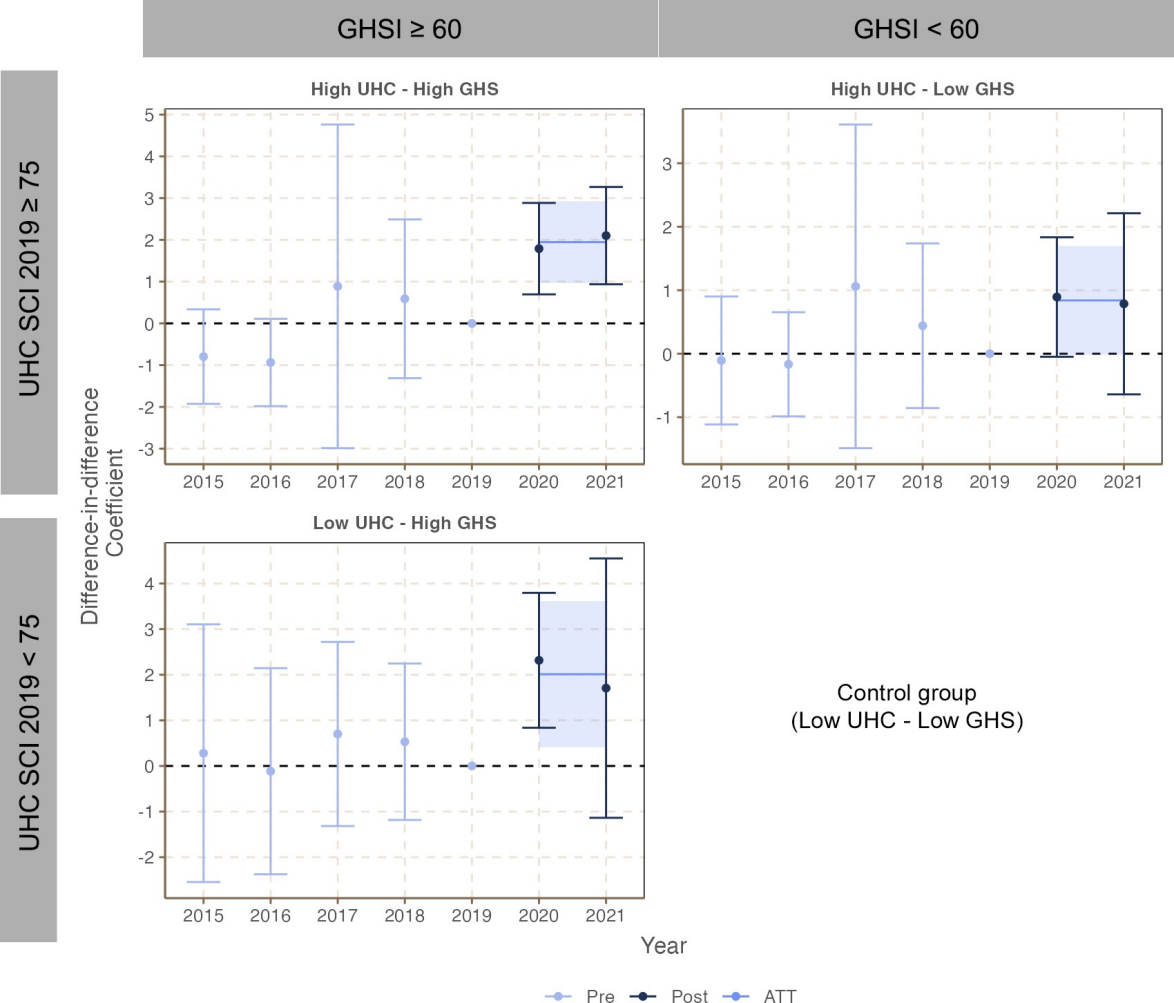
Stratified difference-in-difference results by country categories.

As summarized in Table 3 (Models 3~5) and Fig 2 (with full results available in Tables F~H in S1 Text), the results overall suggest a synergy between UHC and GHS capacities. While countries with high GHS appeared to safeguard their vaccination coverage during the pandemic regardless of countries’ UHC capacities, countries with high UHC capacities did not show such protective effects during the pandemic when not supported by high GHS capacities. In summary, these findings empirically demonstrated the complementing and synergistic effects of GHS and UHC capacities, and the importance of GHS capacity for augmenting the protective effect of UHC capacity in safeguarding essential health service delivery during a public health emergency.

The correlation between UHC SCI 2019 and overall vaccination coverage across all vaccine types was 0.37, while the correlation between UHC SCI 2019 and DTP3 and MCV1 was 0.51 and 0.50, respectively. All of the findings were consistent when the analyses were repeated by excluding DTP3 and MCV1 coverage rates from the independent variables (Figs E and F and Tables I~M in S1 Text).

## Discussion

Understanding the determinants of health system resilience against the COVID-19 pandemic is important to bolster our current and future resilience against public health emergencies. The two key global health agendas that have received particular attention from national and global policymakers are UHC and GHS. Building on our previous research [9], this study examined the individual and synergistic effects of UHC and GHS on childhood vaccination rates during the first two years of the COVID-19 pandemic. In line with previous research [7–9], we not only confirmed that progress towards UHC prevented a significant reduction in immunization coverage in 2020, but also showed that this preventive effect was even larger in 2021. Similar to UHC, we found that progress towards GHS also resulted in protective effects, both in 2020 and 2021. This is particularly interesting because previous studies have reported null or mixed associations between GHS and countries’ pandemic resilience [10–13], making it difficult to ascertain the role of GHS during public health emergencies. Our results also indicate that the protective effects of UHC and GHS became more pronounced as time elapsed—specifically, the protective effect sizes were larger in 2021 compared to 2020—which could be explained by UHC and GHS capacities enabling health system recoveries or their protective effects becoming more apparent after emergency measures and funding gradually were eased in 2021.

This study was one of the first studies to examine the potential protective effects associated with the synergy between UHC and GHS. When taken together, our results showed that progress towards UHC alone may not be sufficient to protect countries’ essential immunization service delivery against disruptions due to the COVID-19 pandemic. Contrariliy, progress towards GHS, even in the absence of sufficient UHC capacity, likely prevented declines in immunization coverage rates during the pandemic. Our results also suggest there was a synergistic protective effect resulting from progress towards both health agendas.

In addition to being one of the first studies to examine the synergistic effects of UHC and GHS on population health, this study is novel in that it quantifies the possible effects of countries’ progress towards UHC or GHS on their health system performance during the first two years of the COVID-19 pandemic. Our finding that the effects of both UHC and GHS on health system resilience became more pronounced in 2021 indicates these health agendas and indicators may become more impactful as time elapses, which could be significant for countries’ post-pandemic prioritization of recovery policies.

An important consideration in interpreting the results is the inherent limitations associated with country-level estimates. Although our data was strictly derived from commonly used and publicly available data sources, country-level estimates do not account for subnational variations, differences in data quality, or other biases inherent in such estimates [30, 31]. There are, however, few alternative global health data sources that allow researchers to make cross-country comparisons, especially during the pandemic. Another limitation is the timeframe of our study. While we were able to extend our previous analysis of UHC’s protective effects on vaccination coverage [9] by including data from the second year of the pandemic, more data on post-pandemic years can facilitate an assessment of the long-term effects of UHC and GHS capacities in safeguarding essential health service delivery across countries.

As national and global policymakers set priorities, develop policies, and make investment decisions to combat the ongoing COVID-19 pandemic and bolster the resilience of health systems against future epidemics or pandemics, it is imperative to have empirical evidence on which health policies and agendas enhance health systems resilience. This study found that greater progress towards both UHC and GHS safeguarded essential health service delivery during the first two years of the pandemic, and progress towards GHS alone may be sufficient for yielding a protective effect. Our results emphasized the importance of sustaining strategic investments into both health agendas and call for future research into the possible synergistic effects of the two health agendas on health systems resilience.

Future analyses can be conducted by employing our methodology to assess how the effects of UHC and GHS capacities on health systems resilience change over time and across different regions across the globe. Such research with a longer time horizon has the potential to assist policymakers in identifying policies that are likely to safeguard population health during health emergencies, including pandemics. Finally, as also proposed by other studies [9, 32], other health indicators, such as maternal and neonatal mortality rates, can be used as proxies for essential health service delivery to build a more robust body of evidence given the dearth of research in this area.

## Supporting information

S1 Text (DOCX)
