# Supplementary material for: The synergistic impact of Universal Health Coverage and Global Health Security on health service delivery during the Coronavirus Disease-19 pandemic: A difference-in-difference study of childhood immunization coverage from 192 countries
Source: PLOS Glob Public Health. 2024 May 10;4(5):e0003205. doi: 10.1371/journal.pgph.0003205 (PMC11086828; doi:10.1371/journal.pgph.0003205)
Supplement: S1 Text — (DOCX) [file pgph.0003205.s001.docx]

**The synergistic impact of Universal Health Coverage and Global Health Security on health service delivery during the Coronavirus Disease-19 pandemic: A difference-in-difference study of childhood immunization coverage from 192 countries**

**S1 Text**

1. Additional text on the assumptions of difference-in-difference analysis

Our main hypothesis was that countries’ greater progress towards UHC and/or GHS capacities enable countries’ health systems to be more resilient to external shocks, such as the COVID-19 pandemic. This resilience will affect countries’ abilities to provision basic and essential public health services, including routine childhood immunizaitons. To test this hypothesis, we adopted the concept of a quasi-experimental design by using a difference-in-difference (DiD) analysis.[1] Typical DiD is used to assess the causal effect of the policy or program (intervention) by comparing it with the control group before and after the intervention where a clear temporal cutoff of pre-intervention and post-intervention exists. DiD analyses also need to satisfy three assumptions: 1) a parallel pre-trend of outcomes between intervention and control group prior to the treatment; 2) no external spillover of outcomes across the two groups; and 3) intervention unrelated to outcome at baseline (randomization).[1] We made following assumptions to support our analysis accordingly:

1. Public health crises like the COVID-19 pandemic will increase the overall health system burden, which, in turn, causes disruptions to countries’ routine public health activities such as national immunization programs.
2. Countries’ health system progress towards UHC is associated with the resilience of the healthcare system to public health crises, such as COVID-19 pandemic. When public health crises occur, countries with greater progress towards UHC will experience less disruption to their routine public health activities.
3. In non-crisis situations (pre-COVID-19-pandemic), countries with high levels of UHC and low levels of UHC will show similar pre-trends in immunization coverage (parallel trend).
4. One country’s demand on essential vaccines will not affect other countries’ vaccine supplies due to the reliable procurement, sufficient distribution of essential vaccines across the globe, and the availability of emergency stockpiles (no external spillover).[2-4] This assumption, while necessary to conduct empirical analyses, may not be true in every case as, for instance, high-income countries may prioritize the health of their populations more than low-income countries. Conversely, investments in one country’s health security may have positive spillover effects on other countries as better subnational or national surveillance may lead to disease outbreaks being detected and mitigated prior to becoming an epidemic or pandemic. Should there be a spillover effect in these limited and sometimes unquantifiable situations, we assess, on net, that our predictions will likely be skewed towards the conservative and underestimated direction given the likely positive spillover effects of GHS and UHC onto other countries’ health outcomes. However, this assessment should be further investigated, and attempts should be made to try to quantify the positive and negative spillover effects of various national policies on UHC and GHS in the future.
5. This study’s code (in R) and data are available at: <https://github.com/sk9076/UHC_GHS_2021>

Table A. STROBE Statement—Checklist of items that should be included in reports of ***cross-sectional studies***

|  | **Item No** | **Recommendation** | **Section** |
| --- | --- | --- | --- |
| **Title and abstract** | 1 | (*a*) Indicate the study’s design with a commonly used term in the title or the abstract | Title, Abstract |
|  |  | (*b*) Provide in the abstract an informative and balanced summary of what was done and what was found | Abstract |
| **Introduction** | | |  |
| Background/rationale | 2 | Explain the scientific background and rationale for the investigation being reported | Introduction |
| Objectives | 3 | State specific objectives, including any prespecified hypotheses | Introduction |
| **Methods** | | |  |
| Study design | 4 | Present key elements of study design early in the paper | Methods |
| Setting | 5 | Describe the setting, locations, and relevant dates, including periods of recruitment, exposure, follow-up, and data collection | Methods (Data) |
| Participants | 6 | (*a*) Give the eligibility criteria, and the sources and methods of selection of participants | Methods (Data) |
| Variables | 7 | Clearly define all outcomes, exposures, predictors, potential confounders, and effect modifiers. Give diagnostic criteria, if applicable | Methods (Data, Statistical Analysis) |
| Data sources/ measurement | 8* | For each variable of interest, give sources of data and details of methods of assessment (measurement). Describe comparability of assessment methods if there is more than one group | Methods (Data) |
| Bias | 9 | Describe any efforts to address potential sources of bias | Methods (Data, Statistical Analysis) |
| Study size | 10 | Explain how the study size was arrived at | Methods (Data) |
| Quantitative variables | 11 | Explain how quantitative variables were handled in the analyses. If applicable, describe which groupings were chosen and why | Methods (Data) |
| Statistical methods | 12 | (*a*) Describe all statistical methods, including those used to control for confounding | Methods (Statistical Analysis) |
|  |  | (*b*) Describe any methods used to examine subgroups and interactions | Methods (Statistical Analysis) |
|  |  | (*c*) Explain how missing data were addressed | Methods (Statistical Analysis) |
|  |  | (*d*) If applicable, describe analytical methods taking account of sampling strategy | N/A |
|  |  | (*e*) Describe any sensitivity analyses | N/A |
| **Results** | | |  |
| Participants | 13* | (a) Report numbers of individuals at each stage of study—eg numbers potentially eligible, examined for eligibility, confirmed eligible, included in the study, completing follow-up, and analysed | Results |
|  |  | (b) Give reasons for non-participation at each stage | Results |
|  |  | (c) Consider use of a flow diagram | NA |
| Descriptive data | 14* | (a) Give characteristics of study participants (eg demographic, clinical, social) and information on exposures and potential confounders | Results (Table 1,2) |
|  |  | (b) Indicate number of participants with missing data for each variable of interest |  |
| Outcome data | 15* | Report numbers of outcome events or summary measures | Results (Table 2) |
| Main results | 16 | (*a*) Give unadjusted estimates and, if applicable, confounder-adjusted estimates and their precision (eg, 95% confidence interval). Make clear which confounders were adjusted for and why they were included | Results (Table 3) |
|  |  | (*b*) Report category boundaries when continuous variables were categorized | Results |
|  |  | (*c*) If relevant, consider translating estimates of relative risk into absolute risk for a meaningful time period | N/A |
| Other analyses | 17 | Report other analyses done—eg analyses of subgroups and interactions, and sensitivity analyses | Results (Table 3, Figures 1-2), Supplementary Materials |
| **Discussion** | | |  |
| Key results | 18 | Summarise key results with reference to study objectives | Discussion |
| Limitations | 19 | Discuss limitations of the study, taking into account sources of potential bias or imprecision. Discuss both direction and magnitude of any potential bias | Discussion |
| Interpretation | 20 | Give a cautious overall interpretation of results considering objectives, limitations, multiplicity of analyses, results from similar studies, and other relevant evidence | Discussion |
| Generalisability | 21 | Discuss the generalisability (external validity) of the study results | Discussion |
| **Other information** | | |  |
| Funding | 22 | Give the source of funding and the role of the funders for the present study and, if applicable, for the original study on which the present article is based | N/A |


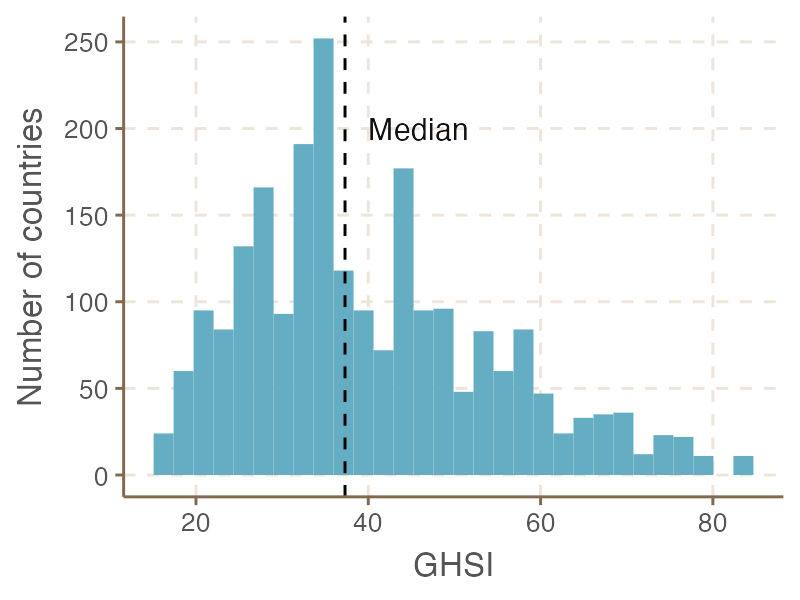


Fig A. Distribution of Global Health Security Index (GHSI) score


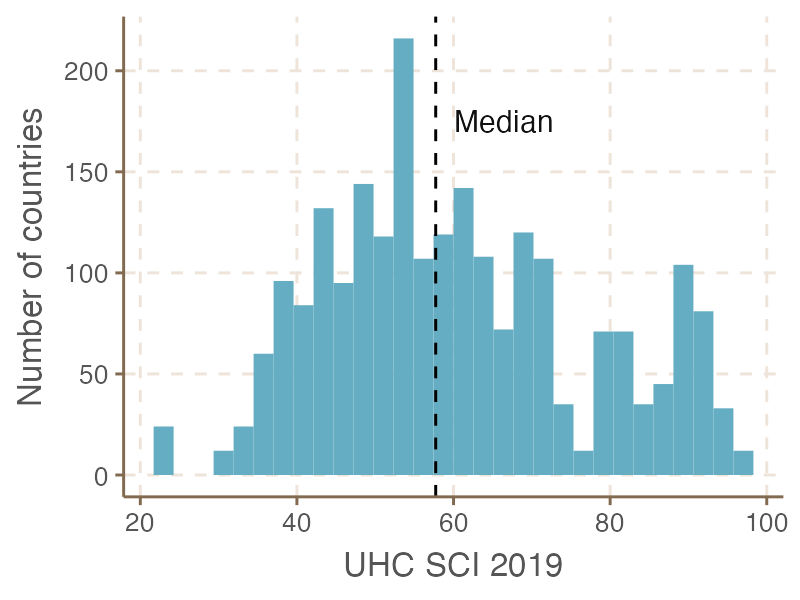


Fig B. Distribution of Universal Health Coverage Service Coverage Index (UHC SCI) 2019 score


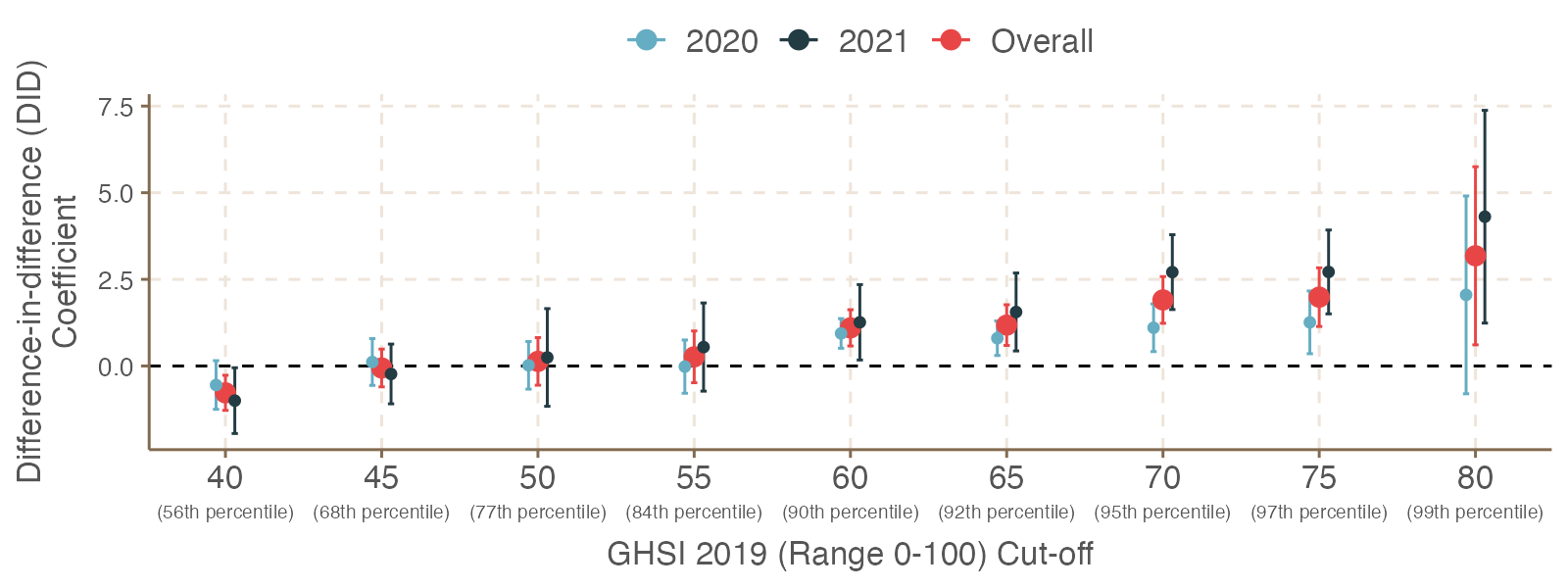


Fig C. Difference-in-difference coefficient based on different GHSI 2019 cutoff values.

Table B. Difference-in-difference coefficient based on different GHSI 2019 cutoff values.

| GHSI 2019 cutoff | Difference-in-difference coefficient (95% Confidence interval) | | | p-value for parallel pre-trend assumption check |
| --- | --- | --- | --- | --- |
|  | Overall | 2020 | 2021 |  |
| 40 | -0.774  (-1.346, -0.203) | -0.549  (-1.259, 0.162) | -1.000  (-1.785, -0.215) | 0.0015 |
| 45 | -0.058  (-0.579, 0.463) | 0.115  (-0.511, 0.740) | -0.230  (-1.042, 0.582) | 0.0553 |
| 50 | 0.133  (-0.589, 0.854) | 0.019  (-0.635, 0.673) | 0.246  (-1.302, 1.794) | 0.0119 |
| 55 | 0.264  (-0.501, 1.029) | -0.019  (-0.729, 0.692) | 0.546  (-0.676, 1.768) | 0.0018 |
| 60 | 1.100  (0.571, 1.629) | 0.939  (0.523, 1.355) | 1.261  (0.148, 2.374) | 0.1069 |
| 65 | 1.180  (0.562, 1.798) | 0.801  (0.330, 1.273) | 1.558  (0.548, 2.568) | 0.2622 |
| 70 | 1.906  (1.213, 2.599) | 1.104  (0.402, 1.805) | 2.708  (1.638, 3.779) | 0.5101 |
| 75 | 1.986  (1.117, 2.856) | 1.259  (0.312, 2.206) | 2.714  (1.502, 3.926) | 0.3715 |
| 80 | 3.180  (0.737, 5.623) | 2.052  (-0.703, 4.807) | 4.308  (1.322, 7.293) | 0.0514 |


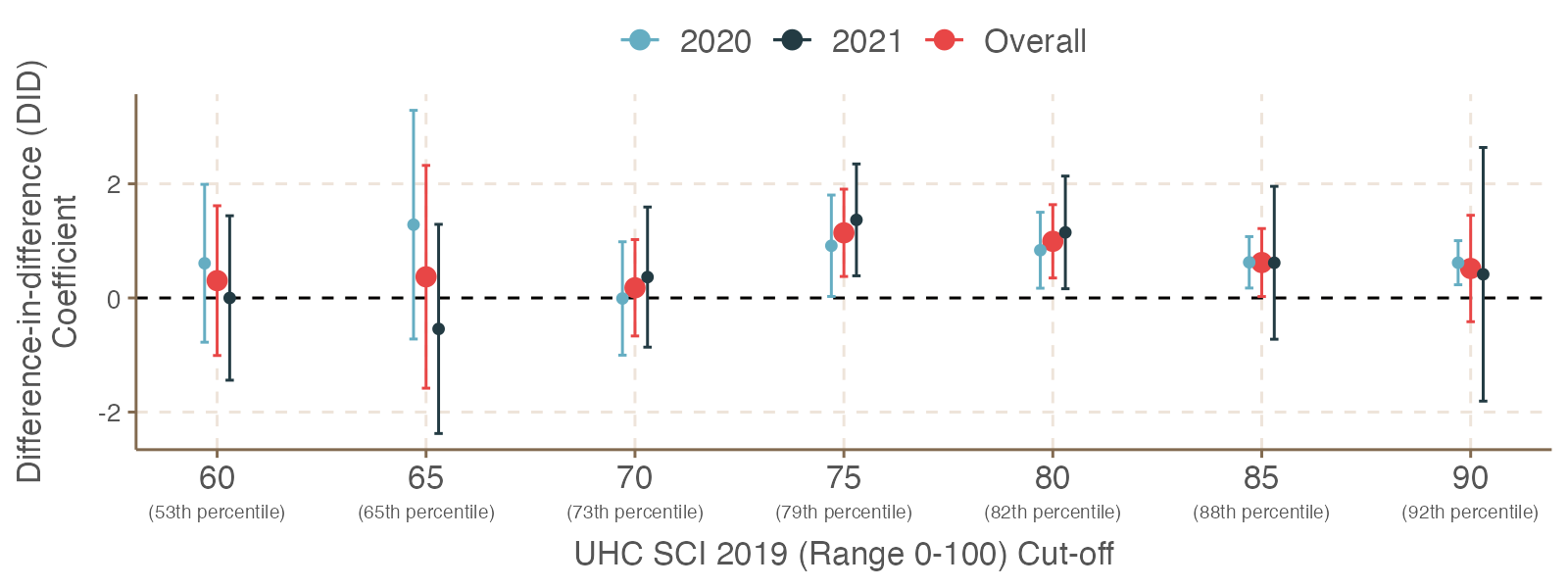


Fig D. Difference-in-difference coefficient based on different UHC SCI 2019 cutoff values.

Table C. Difference-in-difference coefficient based on different UHC SCI 2019 cutoff values.

| UHC SCI 2019 cutoff | Difference-in-difference coefficient (95% Confidence interval) | | | p-value for parallel pre-trend assumption check |
| --- | --- | --- | --- | --- |
|  | Overall | 2020 | 2021 |  |
| 60 | 0.608  (-0.822, 2.037) | 0.000  (-1.595, 1.594) | 0.304  (-0.780, 1.387) | <0.001 |
| 65 | 1.284  (-0.826, 3.394) | -0.543  (-2.424, 1.339) | 0.371  (-1.549, 2.290) | 0.1261 |
| 70 | -0.009  (-1.069, 1.050) | 0.366  (-0.810, 1.541) | 0.178  (-0.700, 1.057) | 0.5514 |
| 75 | 0.915  (0.070, 1.760) | 1.368  (0.294, 2.442) | 1.141  (0.415, 1.868) | 0.5321 |
| 80 | 0.836  (0.129, 1.544) | 1.149  (0.133, 2.165) | 0.993  (0.375, 1.610) | 0.0931 |
| 85 | 0.624  (0.160, 1.088) | 0.616  (-0.670, 1.902) | 0.620  (0.026, 1.215) | 0.0725 |
| 90 | 0.618  (0.224, 1.011) | 0.415  (-1.826, 2.655) | 0.516  (-0.402, 1.435) | 0.1290 |

Table D. Complete results of the difference-in-difference analysis using the GHS (GHSI ≥ 60) (Model 1 in Table 3 of the manuscript).

| Year | Difference-in-difference (DiD) coefficient | 95% Confidence Interval (CI) |
| --- | --- | --- |
| 2015 | -0.189 | (-1.268, 0.891) |
| 2016 | -0.526 | (-1.264, 0.212) |
| 2017 | -0.119 | (-0.944, 0.707) |
| 2018 | -0.203 | (-0.783, 0.376) |
| 2019 | 0 |  |
| 2020 | 0.939 | (0.474, 1.404) |
| 2021 | 1.261 | (0.180, 2.342) |
| Overall (across 2020 and 2021) | 1.100 | (0.570, 1.630) |
| p-value for parallel pre-trend assumption check | 0.107 | |

* All analyses are controlled for countries’ income group, vaccine type, calendar year, and geographical region.

GHSI=Global Health Security Index

Table E. Complete results of the difference-in-difference analysis using the UHC (UHC SCI 2019 ≥ 75) (Model 2 in Table 3 of the manuscript).

| Year | Difference-in-difference (DiD) coefficient | 95% Confidence Interval (CI) |
| --- | --- | --- |
| 2015 | 0.242 | (-0.669, 1.152) |
| 2016 | -0.053 | (-0.906, 0.800) |
| 2017 | 0.991 | (-1.573, 3.554) |
| 2018 | 0.641 | (-0.616, 1.899) |
| 2019 | 0 |  |
| 2020 | 0.915 | (0.020, 1.810) |
| 2021 | 1.368 | (0.365, 2.371) |
| Overall (across 2020 and 2021) | 1.141 | (0.388, 1.895) |
| p-value for parallel pre-trend assumption check | 0.532 | |

* All analyses are controlled for countries’ income group, vaccine type, calendar year, and geographical region.

UHC SCI=Universal Health Coverage Service Coverage Index

Table F. Complete results of the difference-in-difference-in-difference analysis using the UHC SCI 2019 and the GHSI (**UHC SCI 2019**$\boldsymbol{\geq}$ **75 and GHSI**$\boldsymbol{\geq}$**60)** (Model 3 in Table 3 of the manuscript).

| Year | Difference-in-difference (DiD) coefficient | 95% Confidence Interval (CI) |
| --- | --- | --- |
| 2015 | -0.795 | (-1.927, 0.336) |
| 2016 | -0.936 | (-1.983, 0.110) |
| 2017 | 0.887 | (-2.988, 4.763) |
| 2018 | 0.589 | (-1.312, 2.490) |
| 2019 | 0.000 |  |
| 2020 | 1.790 | (0.695, 2.885) |
| 2021 | 2.102 | (0.937, 3.267) |
| Overall (across 2020 and 2021) | 1.946 | (0.968, 2.924) |
| p-value for parallel pre-trend assumption check | 0.106 | |

* All analyses are controlled for countries’ income group, vaccine type, calendar year, and geographical region.

** Control group for DiD includes countries with UHC SCI 2019<75 and GHSI<60.

UHC SCI=Universal Health Coverage Service Coverage Index; GHSI=Global Health Security Index

Table G. Complete results of the difference-in-difference-in-difference analysis using the UHC SCI 2019 and the GHSI (**UHC SCI 2019**$\boldsymbol{\geq}$**75 and GHSI**$\boldsymbol{<}$**60)** (Model 4 in Table 3 of the manuscript).

| Year | Difference-in-difference (DiD) coefficient | 95% Confidence Interval (CI) |
| --- | --- | --- |
| 2015 | 0.207 | (-0.843, 1.257) |
| 2016 | -0.106 | (-1.134, 0.922) |
| 2017 | 1.003 | (-2.209, 4.215) |
| 2018 | 0.642 | (-0.839, 2.124) |
| 2019 | 0 |  |
| 2020 | 0.684 | (-0.302, 1.67) |
| 2021 | 1.055 | (-0.196, 2.307) |
| Overall (across 2020 and 2021) | 0.869 | (0.023, 1.716) |
| p-value for parallel pre-trend assumption check | 0.107 | |

* All analyses are controlled for countries’ income group, vaccine type, calendar year, and geographical region.

** Control group for DiD includes countries with UHC SCI 2019<75 and GHSI<60.

UHC SCI=Universal Health Coverage Service Coverage Index; GHSI=Global Health Security Index

Table H. Complete results of the difference-in-difference-in-difference analysis using the UHC SCI 2019 and the GHSI (**UHC SCI 2019**$\boldsymbol{<}$**75 and GHSI**$\boldsymbol{\geq}$**60)** (Model 5 in Table 3 of the manuscript).

| Year | Difference-in-difference (DiD) coefficient | 95% Confidence Interval (CI) |
| --- | --- | --- |
| 2015 | -0.189 | (-1.163, 0.786) |
| 2016 | -0.526 | (-1.235, 0.182) |
| 2017 | -0.119 | (-0.952, 0.714) |
| 2018 | -0.203 | (-0.746, 0.339) |
| 2019 | 0 |  |
| 2020 | 0.939 | (0.527, 1.351) |
| 2021 | 1.261 | (0.25, 2.271) |
| Overall (across 2020 and 2021) | 1.1 | (0.579, 1.621) |
| p-value for parallel pre-trend assumption check | 0.670 | |

* All analyses are controlled for countries’ income group, vaccine type, calendar year, and geographical region.

** Control group for DiD includes countries with UHC SCI 2019<75 and GHSI<60.

UHC SCI=Universal Health Coverage Service Coverage Index; GHSI=Global Health Security Index


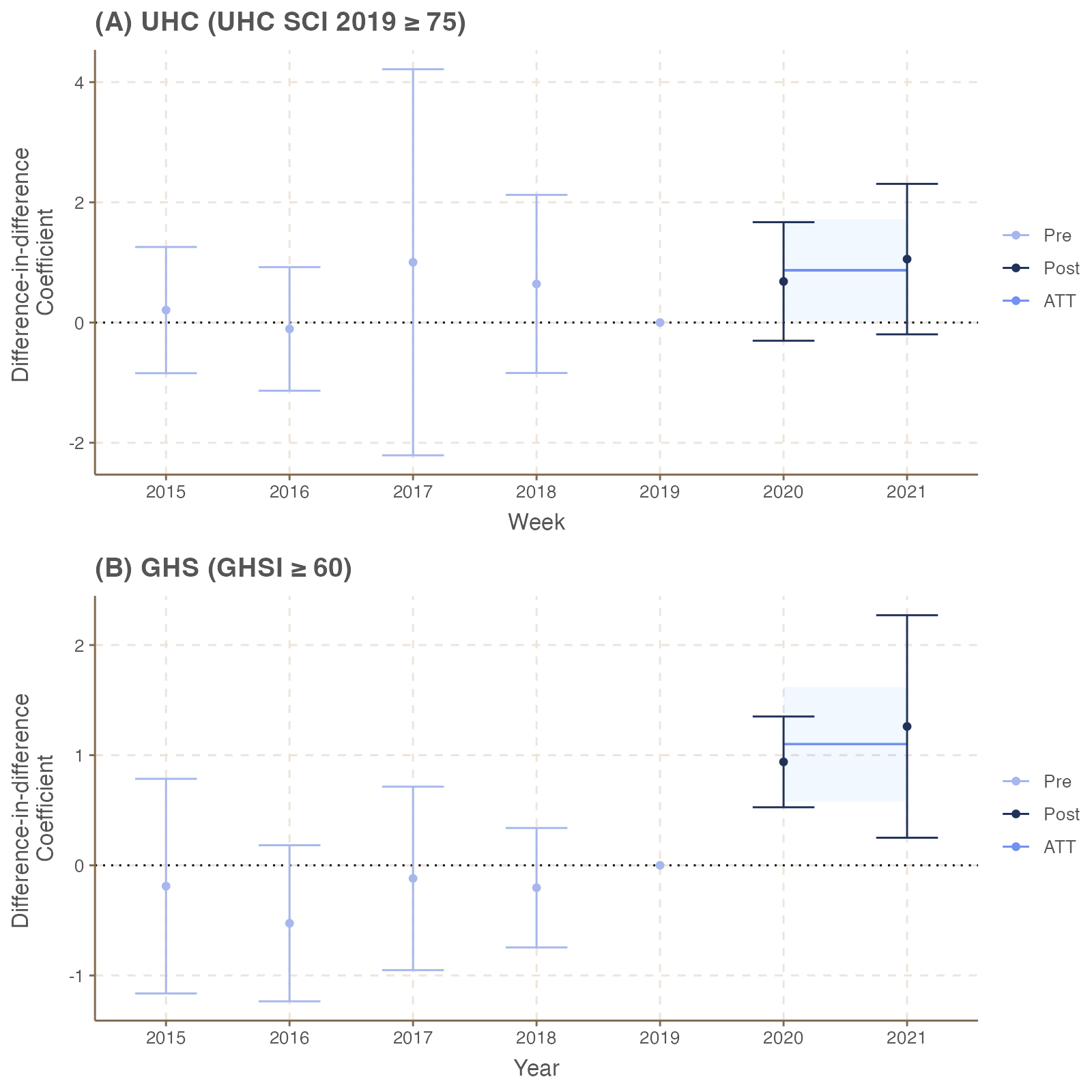


Fig E. Difference-in-difference model results across 2020 and 2021excluding DTP3 and MCV1.

Table I. Complete results of the difference-in-difference analysis using the GHS (GHSI ≥ 60) excluding DTP3 and MCV1.

| Year | Difference-in-difference (DiD) coefficient | 95% Confidence Interval (CI) |
| --- | --- | --- |
| 2015 | -0.189 | (-1.268, 0.891) |
| 2016 | -0.526 | (-1.264, 0.212) |
| 2017 | -0.119 | (-0.944, 0.707) |
| 2018 | -0.203 | (-0.783, 0.376) |
| 2019 | 0 |  |
| 2020 | 0.939 | (0.474, 1.404) |
| 2021 | 1.261 | (0.180, 2.342) |
| Overall (across 2020 and 2021) | 1.100 | (0.570, 1.630) |
| p-value for parallel pre-trend assumption check | 0.107 | |

* All analyses are controlled for countries’ income group, vaccine type, calendar year, and geographical region.

GHSI=Global Health Security Index

Table J. Complete results of the difference-in-difference analysis using the UHC (UHC SCI 2019 ≥ 75) excluding DTP3 and MCV1.

| Year | Difference-in-difference (DiD) coefficient | 95% Confidence Interval (CI) |
| --- | --- | --- |
| 2015 | 0.242 | (-0.669, 1.152) |
| 2016 | -0.053 | (-0.906, 0.800) |
| 2017 | 0.991 | (-1.573, 3.554) |
| 2018 | 0.641 | (-0.616, 1.899) |
| 2019 | 0 |  |
| 2020 | 0.915 | (0.020, 1.810) |
| 2021 | 1.368 | (0.365, 2.371) |
| Overall (across 2020 and 2021) | 1.141 | (0.388, 1.895) |
| p-value for parallel pre-trend assumption check | 0.532 | |

* All analyses are controlled for countries’ income group, vaccine type, calendar year, and geographical region.

UHC SCI=Universal Health Coverage Service Coverage Index


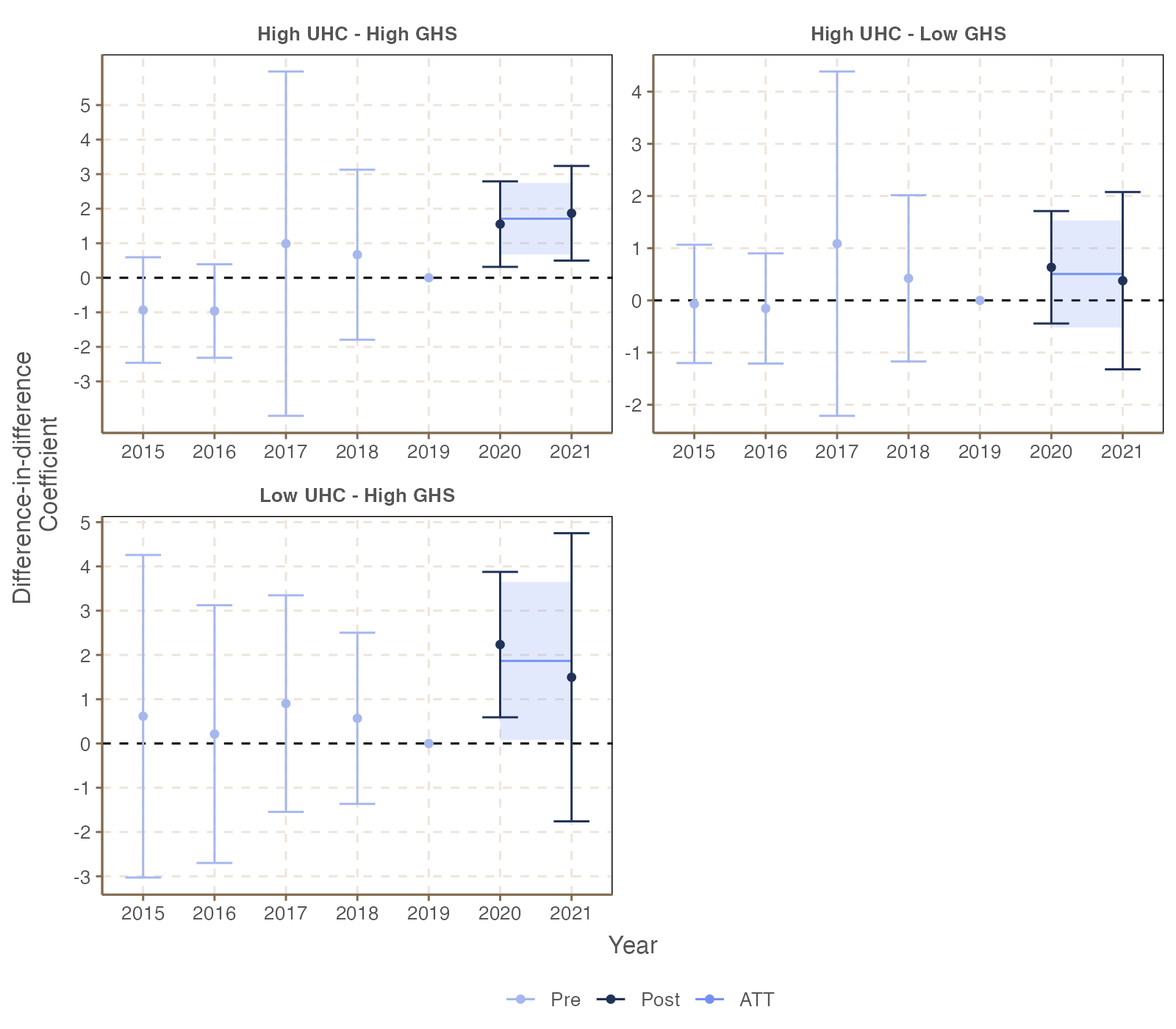


Figure F. Difference-in-difference-in-difference model results by country categories excluding DTP3 and MCV1.

Table K. Complete results of the difference-in-difference-in-difference analysis using the UHC SCI 2019 and the GHSI (**UHC SCI 2019**$\boldsymbol{\geq}$**75 and GHSI**$\boldsymbol{\geq}$**60)** excluding DTP3 and MCV1.

| Year | Difference-in-difference (DiD) coefficient | 95% Confidence Interval (CI) |
| --- | --- | --- |
| 2015 | -0.934 | (-2.463, 0.594) |
| 2016 | -0.962 | (-2.317, 0.392) |
| 2017 | 0.988 | (-3.997, 5.973) |
| 2018 | 0.667 | (-1.794, 3.128) |
| 2019 | 0.000 |  |
| 2020 | 1.554 | (0.316, 2.791) |
| 2021 | 1.867 | (0.497, 3.237) |
| Overall (across 2020 and 2021) | 1.710 | (0.676, 2.745) |
| p-value for parallel pre-trend assumption check | 0.194 | |

* All analyses are controlled for countries’ income group, vaccine type, calendar year, and geographical region.

** Control group for DiD includes countries with UHC SCI 2019<75 and GHSI<60.

UHC SCI=Universal Health Coverage Service Coverage Index; GHSI=Global Health Security Index

Table L. Complete results of the difference-in-difference-in-difference analysis using the UHC SCI 2019 and the GHSI (**UHC SCI 2019**$\boldsymbol{\geq}$**75 and GHSI**$\boldsymbol{<}$**60)** excluding DTP3 and MCV1.

| Year | Difference-in-difference (DiD) coefficient | 95% Confidence Interval (CI) |
| --- | --- | --- |
| 2015 | -0.067 | (-1.200, 1.066) |
| 2016 | -0.155 | (-1.210, 0.901) |
| 2017 | 1.087 | (-2.212, 4.386) |
| 2018 | 0.423 | (-1.170, 2.016) |
| 2019 | 0.000 |  |
| 2020 | 0.634 | (-0.443, 1.711) |
| 2021 | 0.378 | (-1.321, 2.076) |
| Overall (across 2020 and 2021) | 0.506 | (-0.520, 1.533) |
| p-value for parallel pre-trend assumption check | 0.750 | |

* All analyses are controlled for countries’ income group, vaccine type, calendar year, and geographical region.

** Control group for DiD includes countries with UHC SCI 2019<75 and GHSI<60.

UHC SCI=Universal Health Coverage Service Coverage Index; GHSI=Global Health Security Index

Table M. Complete results of the difference-in-difference-in-difference analysis using the UHC SCI 2019 and the GHSI (**UHC SCI 2019**$\boldsymbol{<}$ **75 and GHSI**$\boldsymbol{\geq}$**60)** excluding DTP3 and MCV1.

| Year | Difference-in-difference (DiD) coefficient | 95% Confidence Interval (CI) |
| --- | --- | --- |
| 2015 | 0.615 | (-3.029, 4.259) |
| 2016 | 0.212 | (-2.700, 3.124) |
| 2017 | 0.901 | (-1.546, 3.348) |
| 2018 | 0.569 | (-1.365, 2.502) |
| 2019 | 0.000 |  |
| 2020 | 2.234 | (0.591, 3.877) |
| 2021 | 1.496 | (-1.759, 4.751) |
| Overall (across 2020 and 2021) | 1.865 | (0.081, 3.649) |
| p-value for parallel pre-trend assumption check | 0.560 | |

* All analyses are controlled for countries’ income group, vaccine type, calendar year, and geographical region.

** Control group for DiD includes countries with UHC SCI 2019<75 and GHSI<60.

UHC SCI=Universal Health Coverage Service Coverage Index; GHSI=Global Health Security Index

**References**

1. Fredriksson A, Oliveira GMd. Impact evaluation using Difference-in-Differences. RAUSP Management Journal. 2019;54(4):519-32.

2. UNICEF. Vaccine supplies [Available from: <https://www.unicef.org/supply/vaccines>.

3. World Health Organization. Essential Programme on Immunization 2021 [Available from: <https://www.who.int/teams/immunization-vaccines-and-biologicals/essential-programme-on-immunization>.

4. Yen C, Hyde TB, Costa AJ, Fernandez K, Tam JS, Hugonnet S, et al. The development of global vaccine stockpiles. The Lancet Infectious Diseases. 2015;15(3):340-7.
